# Acne Detection by Ensemble Neural Networks

**DOI:** 10.3390/s22186828

**Published:** 2022-09-09

**Authors:** Hang Zhang, Tianyi Ma

**Affiliations:** 1 School of Materials Science and Engineering, Nanyang Technological University, Singapore 639798, Singapore; 2 Nanjing MetaEntropy Intelligent Technology Co., Ltd., Nanjing 210030, China

**Keywords:** acne detection, ensemble model, acne severity, acne position

## Abstract

Acne detection, utilizing prior knowledge to diagnose acne severity, number or position through facial images, plays a very important role in medical diagnoses and treatment for patients with skin problems. Recently, deep learning algorithms were introduced in acne detection to improve detection precision. However, it remains challenging to diagnose acne based on the facial images of patients due to the complex context and special application scenarios. Here, we provide an ensemble neural network composed of two modules: (1) a classification module aiming to calculate the acne severity and number; (2) a localization module aiming to calculate the detection boxes. This ensemble model could precisely predict the acne severity, number, and position simultaneously, and could be an effective tool to help the patient self-test and assist the doctor in the diagnosis.

## 1. Introduction

Computer vision [1,2] is a simulation of biological vision by utilizing the computer and relevant equipment. The core aim is to extract the desired information from the target pictures and videos. With the rapid development of deep learning technology, the knotty tasks in computer vision can be resolved with high precision by utilizing novel algorithms [3,4,5,6], such as convolutional neural networks, long short-term memory networks, recurrent neural networks, etc. Various network architectures (e.g., AlexNet, VGGNet, ResNet, MobileNet, etc.) have been proposed to “read” the pictures and are widely used as the backbone in diverse applications of computer vision. Usually, depending on the different application scenarios, computer vision can be roughly divided into three subfields, i.e., visual recognition, visual tracking, and image restoration. Visual recognition [7,8,9,10,11,12,13,14,15,16,17,18,19], one of the hottest research fields among them, has been widely concerned due to its significant applications in our daily life. Wu et al. divided the recognition problems into four fundamental tasks [16] (i.e., image classification, object detection, instance segmentation and semantic segmentation) based on their various mission content. Chai et al. introduced their applications in different scenarios in detail [1]. Even more to the point, visual recognition can not only be used in traditional computer vision tasks, such as image restoration [20,21,22], image stitching [23,24,25] and face recognition [26,27,28], but also shows significant applications in various engineering fields, including material analyses [29,30,31], material synthesis [32,33,34], metamaterial design [35,36,37], etc.

Considering their good capabilities of extracting information from pictures, the technologies of visual recognition have also been used in healthcare to help doctors diagnose diseases, especially skin diseases whose visual representations are easier to spot. Since skin health can be easily affected by the living environment and lifestyle, people who live with unhealthy habits (e.g., smoking, excessive sun exposure, sleeping in a humid environment) are more prone to skin problems. Note that although the probability of skin diseases is not related to regions and ages, skin diseases [38,39,40,41,42,43] are one of the common human diseases and cause many people anxiety and depression. However, diagnosing a skin disease is very challenging and depends highly on the experience of dermatologists. Many patients cannot even get a professional diagnosis due to the shortage of dermatologists. As we all know, timely and accurate diagnosis is significant for treating skin diseases. By utilizing deep learning and computer vision, more patients could get instant assessment and proper treatment. For example, Liu et al. put forward a deep learning system to assist general practitioners in diagnosing skin conditions [40]. Srinivasu et al. combined the MobileNet V2 and LSTM to classify skin disease and the proposed model shows better performance in tumor classification and progress analysis [42].

This paper investigates how to use deep learning to diagnose facial acne, a common skin disease. The occurrence of acne is closely related to excessive sebum secretion, blockage of the sebaceous duct, bacterial infection and inflammatory reaction. Since there are many types of facial acne, it is quite challenging to design an expert system to diagnose all these types of facial acne. As a preliminary work on computer-aided diagnoses of facial acne, we aim to evaluate the severity and locate the acne according to the facial images. In this paper, we propose an ensemble model to assess the acne severity, numbers and positions of the facial images simultaneously in the inference. Compared with the previous research regarding acne detection through neural networks: (1) we improve the prediction accuracy in the number and severity of face acne by dataset reclassification and random sampling; (2) we introduce a localization module to predict the location of facial acne. Guided by the extracted features in the classification modules, the model here could precisely calculate the acne position, while previously reported models can hardly predict the acne severity, number and location simultaneously.

## 2. Related Work

In this section, we will introduce several representative studies about the diagnoses of facial acne through deep learning.

### 2.1. Acne Grading

Acne grading [44,45,46,47,48,49,50], a specific application of image classification, aims to estimate the severity of facial acne based on facial images. Previous works mainly take the acne severity as the label and use neural networks to classify the severity. Specifically, Shen et al. utilized two classifiers (i.e., binary classifier and septenary classifier) to diagnose facial acne automatically [48]. They divided acne into seven categories, including papule, cyst, blackhead, normal skin, pustule, whitehead and nodule. The binary classifier could distinguish whether the image consists of skin patches based on the features extracted by the pertained VGG16. The septenary classifier has a similar network structure to the binary classifier and could output the probability of each acne class. In 2019, Zhao et al. developed a lightweight model to assess the acne severity of selfie images taken by mobile phones, greatly reducing the requirements for image resolution. They divided each face image into four skin patches, corresponding to the forehead, right cheek, left cheek and chin by utilizing OpenCV and adopted a new image rolling augmentation approach to improve the spatial sensitivity of CNN models. Similarly, Yang et al. split the clinical images into four regions and constructed a deep learning model to assess the acne severity of each clinical image [45].

Note that the above models need complex image preprocessing, including dividing the whole face region into several regular subregions according to the features in the images. Researchers also use the whole image as the input to simplify the evaluation process. In 2019, Lim et al. developed an automatic system to calculate the Investigator’s Global Assessment (i.e., IGA) scale [49], a criterion for measuring acne severity. In the IGA scale, there are a total of five levels from 0 to 4, corresponding to clear, almost clear, mild, moderate and severe, respectively. Due to the limited numbers of training images, the authors simplified the five levels into three groups (i.e., 0–1, 2, 3–4) and used data augmentation (i.e., cropping, contrast adjustment, intensity scaling and shifting/scaling) to generate images similar to the real image. The total number of training images increases to more than 6000, about 20 times that of the original. As for the network architectures, the authors adopted three high-performing convolution neural networks (i.e., DenseNet, Inception v4 and ResNet18), and all are trained separately from scratch on three image sizes. They concluded that the Inception v4 model outperforms the other two models and the best classification accuracy is 67%. To solve the problem of insufficient training data, Wu et al. collected a new dataset ACNE04, which provided the annotations of acne severity and the bounding boxes of lesions [47]. Specifically, the severity was graded by expert dermatologists based on the photograph of half of the face. All of the photographs were taken following the Hayashi grading criterion [51], and taken at an approximate 70-degree angle from the front of the patient. Then, the expert dermatologists manually counted the amount of acne and marked the location of the acne by rectangle boxes. Typically, the acne appeared as a cone, and each “cone” was labeled by a rectangle box, with the apex of the cone approximately in the center of the box. The mark boxes would overlap if some acne was very near to each other. Finally, the amount of acne was counted and the facial images were classified into different acne severities based on the Hayashi grading criterion (i.e., 0–5 for mild, 6–20 for moderate, 21–25 for severe and more than 50 for very severe in half of the face) [51] Different from the previous single-label learning in acne grading, the authors used the Gaussian function to convert each label value into a Gaussian distribution, where the peak was just at the label value. The label distribution of each image can be taken as the probability distribution of the labels after normalization. Firstly, the resized facial image was encoded into a feature vector via ResNet-50. Then, two regression layers were added to calculate the label (i.e., lesion numbers and acne severity) distribution of the image. Note that the acne severity depends on the number of lesions; the severity distribution can also be calculated through the softmax operation. Lastly, KL loss is adopted to calculate the loss of the three outputs. To further improve the prediction accuracy in acne severity, Liu et al. proposed a novel ensemble classification framework (i.e., AcneGrader) to classify the acne severity [52]. They utilized the results of various base models as the new feature set, and a customized classifier was then utilized to calculate the acne severity based on the ensemble features. Compared with the previous acne grading method, this model showed a higher performance (e.g., prediction accuracy >85%) and was able to provide accurate diagnoses for patients.

### 2.2. Acne Detection

To locate acne in facial images to assist the doctor in diagnosis, Rashataprucksa et al. utilized Faster-RCNN and R-FCN to train an acne detection model [44]. Precision, recall and mean average precision are utilized to measure the performance of the models. They concluded that R-FCN performed reasonably well with an mAP of up to 28.3%. Similarly, Sangha et al. used the model YOLOv5, which has been pre-trained on the COCO dataset and fine-tune the model on the publicly available dataset ACNE04. The model has good performance in single-class (i.e., acne) detection while showing relative poor performance in multi-class (i.e., severity levels from 1 to 4) detection. Inconsistent illumination, variation in scales and high-density distribution would also bring great challenges to the high-precision acne detection. Min et al. proposed a novel acne detection network named ACNet and achieved prior performance on the ACNE04 dataset [50]. Specifically, the ACNet is composed of Composite Feature Refinement, Dynamic Context Enhancement and Mask-Aware Multi-Attention. The Composite Feature Refinement is composed of two backbone architectures and three feature refinement modules which connect these two backbones at three different levels, such that it could effectively extract the features in the images. The Dynamic Context Enhancement is composed of a feature resizing module and dynamic feature fusion module. It utilizes the multi-scale feature maps from Composite Feature Refinement to remove the scale variation. The Mask-Aware Multi-Attention is composed of a streamlined inception network, mask attention block and context attention block. This part could detect the acne of various sizes by reducing the excessive noise. Compared with previous networks [44] proposed by Rashataprucksa et al, this model shows better acne detection performance (mAP: 20.5) on the ACNE04 dataset.

## 3. Materials and Methods

**Figure 1:**
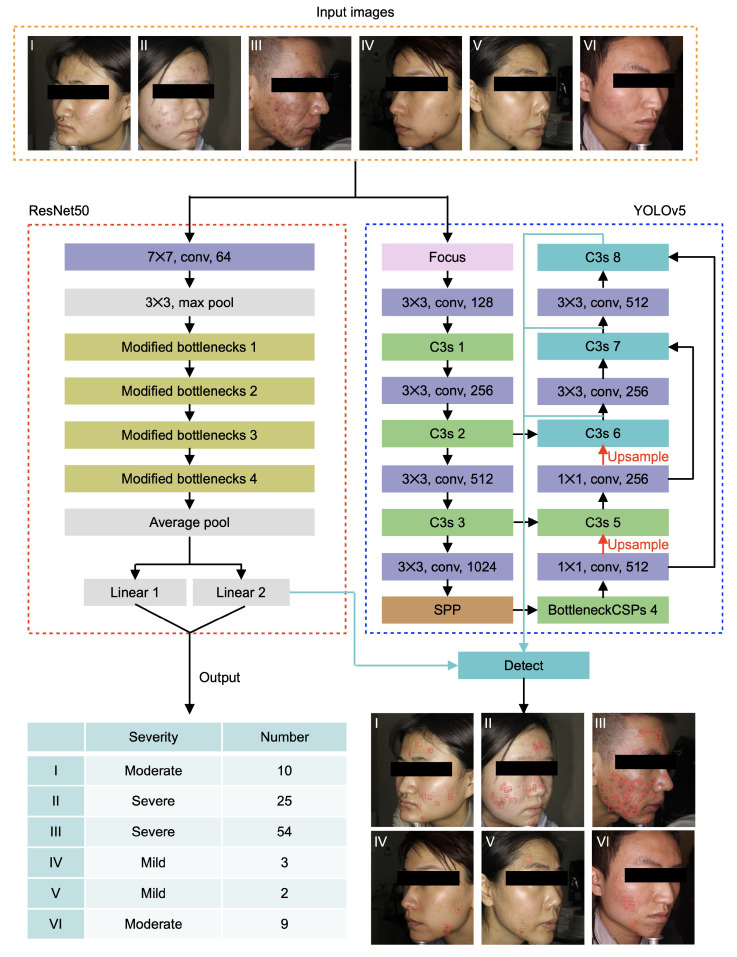
Network architecture of the ensemble neural networks. The ensemble model consists of two submodules, responsible for the severity classification and acne localization. The backbones of the classification and localization module are ResNet50 and YOLOv5, respectively. The end of the classification module connects the localization module, so that the accuracy of acne detection can be improved through combinatorial inference.

Inspired by the previous work on acne grading and detection, we propose an ensemble network (Figure 1) to assess the acne severity and number (i.e., classification module) and to localize the ance position (i.e., localization module) based on the public dataset ACNE04. The following subsections introduce the dataset, network architectures and relevant operations.

### 3.1. Data Preparation

**Figure 2:**
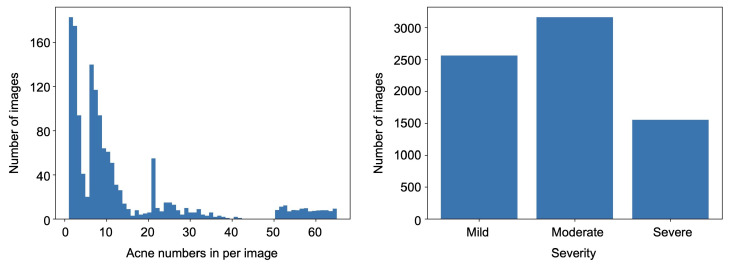
Data distribution in the ACNE04 dataset. There are 1457 images in total and the acne numbers in each image range from 1 to 65.

ACNE04 [47] is a public dataset on facial acne collected by Wu et al., in total providing 1457 facial images of various sizes as well as the corresponding acne severity and number of each image. Additionally, each lesson in the image is marked with a rectangular box by professional dermatologists with a rectangular box. Figure 2 provides the detailed data distribution of the ACNE04 dataset. The maximum acne number in each image is 65, while the minimum number is 1. However, the sample distribution in the dataset is very uneven. For example, a large number of samples gather in the categories with lower acne number (e.g., <10), while there are few samples with acne number from 40 to 50. Specifically, in the categories with acne number 1 and 2, the number of images can reach more than 160. Among these 65 categories, there are only four categories where the number of images is more than 100. In the categories with acne number from 43 to 50, there are only one/two images in each category. The highest difference in the acne number between different categories is more than 160 times, greatly improving the difficulty of model training and evaluation. Inspired by Wu et al. [47], we reclassified the severity classification into three classes to deal with the problem of small sample numbers in the category of high acne numbers. Specifically, when the acne number in an image are between 1 and 5 (including 1 and 5), we set the severity as “mild”; when the acne number is greater than 5 but not greater than 20, we set the severity as “moderate”; when the acne number is greater than 20, we set the severity as “severe”. As shown in the right panel of Figure 2, the second class has the most facial images, while the third class has the least, and the quantity ratio among them is below 2.

Note that predicting the acne number in each image is one of the three tasks (i.e., predicting the acne severity, number and position) in the ensemble model. Thus, smoothing the sample distributions under different acne numbers is very important to the model training. Specifically, we fix the image number (i.e., *N*) in each category. Then, we randomly choose *N* images in the categories with a large sample size (i.e., >*N*), and duplicate the images in the categories with a small sample size (i.e., <*N*). It is worth mentioning that resize operation and normalization are applied to the input images to meet the requirement of the network input.

### 3.2. Classification Module

Each patient can only correspond to one category, that is, each facial image has its category of acne number/severity. In addition to the acne characteristics, the facial images also contain lots of other characteristics, such as face contours, color, brightness, etc. Since these contexts show a great difference between patients, the classification of facial acne images is a computer-vision task with quantities of redundant information. We need to use a deep neural network to eliminate the useless face features and extract the key acne features. Furthermore, to address the vanishing gradient problem during training, we adopt ResNet50 with skip connections as the backbone. Meanwhile, we utilize the bottleneck structure to reduce the feature channels, thus decreasing the parameters amount in the model.

As shown in the red boxes in Figure 1, the backbone of the classification module is ResNet50. Specifically, in this model, a large convolution kernel with the size of 7×7 is utilized to downsample the input images while preserving the original image information as much as possible, and the channels of the input images increase to 64. Then, a max pool is adopted to remove the redundant information. The preprocessed image information is decoded by four modified bottleneck blocks. Each block is composed of three convolution layers. Next, we utilize the average pool to smooth the extracted features and express them as vectors. Lastly, we apply two linear transforms to the feature vectors and output the prediction of acne severities and numbers. Note that batch normalization and ReLU operators are added after each convolutional layer. The main architecture of the classification module is shown in the left panel of Figure 1. The inputs are the images of the patient’s side face and no extra pre-processing is needed. Different from Wu et al. [47], we adopt three different levels (i.e., mild, moderate and severe) to describe the acne severity according to the acne numbers in each image.

The design and selection of loss functions plays a critical role in training neural network. Here, we try several different loss functions and analyze their influence on the model training in detail.

(1) Considering that the main task here is to calculate the acne severity and number, we adopt a cross-entropy loss function, which is very useful when training a classification problem with several classes. The loss function can be written as:
  (1) $$loss_{CEL}=-\frac{1}{N}\sum_{i=1}^{N}\sum_{j=1}^{M}w_{j}y_{ij},$$
  where *N* denotes the total image numbers and *M* denotes the total class numbers (i.e., 4 and 65 for severity and number, respectively). Coefficient $w_{j}$ is a rescaling weight given to each category and is particularly useful when the data distribution is very uneven. Characters $x_{ij}$ and $y_{ij}$ denote the calculated and true probability that the image *i* belongs to category *j*, respectively. Typically, $y_{ij}$ is a Kronecker-like function and can be written as:
  (2) $$y_{i,j}=\left\{\begin{array}{cc} 1, & \;\mathrm{if}\;\mathrm{image}\;i\;\mathrm{in}\;\mathrm{category}\;j;\\ 0, & \;\mathrm{if}\;\mathrm{not}.\; \end{array}\right..$$
(2) Although the number of images in different severity classes is similar, the sample size varies widely in the categories with different acne numbers. Focal loss [53], a loss function aiming to handle the problem of category imbalance, would be helpful for the prediction of acne numbers. Similar to the cross-entropy loss, focal loss tries to make the model pay more attention to the samples, which are hard to classify by changing the sample weights. Furthermore, the function expression can be written as:
  (3) $$\begin{array}{c} loss_{FL}=-\frac{1}{N}\sum_{i=1}^{N}\sum_{j=1}^{M}w_{j}{\left(1-x_{ij}\right)}^{\alpha }y_{ij}\log x_{ij}, \end{array}$$
  where α is a manual parameter named the focusing parameter, which is not smaller than 0. Different from the cross-entropy loss, the additional coefficient ${\left(1-x_{ij}\right)}^{\alpha }$, named the modulating factor, could effectively reduce the loss contribution from the samples which are easy to classify. Note that when α is equal to zero, the focal loss will degenerate to the cross-entropy loss. Specifically, when image *i* does not belong to categories *j*, $x_{ij}$ would be small while the modulating factor is close to 1, showing little influence on the loss. On the contrary, if the image is well classified, $x_{ij}$ would be close to 1, and the modulating factor becomes very small. By tuning the hyperparameters $w_{j}$ and α, we can optimize the training process of the model.
(3) Another common strategy is to transform this classification problem into a regression problem. Inspired by label distribution learning [54,55,56,57,58,59,60,61,62], Wu et al. introduced Kullback–Leibler divergence loss to train the ResNet50 [47]. The general expression of the loss function can be written as:
  (4) $$\begin{array}{c} loss_{KLDL}=-\frac{1}{N}\sum_{i=1}^{N}y_{i}\cdot \left(\log y_{i}-x_{i}\right), \end{array}$$
  where $x_{i}$ and $y_{j}$ are calculated and predicted continuous probability distribution of the image category. Note that the Kullback–Leibler divergence loss can be taken as a variant of the traditional cross-entropy loss. For example, if the probability is strictly set to zero when image *i* does not belong to category *j*, probability distribution $y_{j}$ would become a one-hot vector. We adopt the Normal distribution to generate the label distribution, and the expression can be written as:
  (5) $$\begin{array}{c} f\left(x\right)=\frac{1}{\sqrt{2\pi }\sigma }\exp\left(-\frac{{\left(x-\mu \right)}^{2}}{2\sigma^{2}}\right), \end{array}$$
  where the expectation μ is the true category number and the standard deviation σ is set to 3.

### 3.3. Localization Module

Compared with the classical task of image detection, acne detection in the facial images is a tough detection task, where feature boundaries between different categories are unclear. Specifically, for two images with adjacent acne numbers, they show similar acne characteristics, though they have different facial appearances. These acne characteristics are very small compared with face contours, and can easily be overwhelmed by those large features. It is quite difficult to localize small features with high similarity in different backgrounds. In this paper, we adopt YOLOv5 as the backbone of the localization module. The Focus structure in the backbone would improve the receptive fields and ensure no missing context. The CSP structure (i.e., Cross Stage Partial) could deal with the problem of gradient vanishing during extracting the deep features in the facial images. The SPP (i.e., Spatial Pyramid Pooling) structures could improve the capability of detecting tiny objects.

The operation process of the localization module is demonstrated in the right panel of Figure 1. The inputs are the facial images from different patients, while the outputs are the detection boxes of the acne in the images. We adopt YOLOv5 (blue box in Figure 1) as the backbone of the localization module. Specifically, the architecture of YOLOv5 is composed of four types of convolutional blocks, including Focus block, Conv block, C3 block and SPP block. Firstly, the input images are downsampled by Focus block with increased image channels, so that the image could be resized without loss of information. Then, deeper features are extracted by a series of Conv blocks and C3 blocks. The Conv block consists of a 2D convolutional operator, a batch normalization operator, and a SiLU operator. The C3 block consists of three Conv blocks and several Bottleneck blocks. The SPP (i.e., Spatial Pyramid Pooling) block is inserted in the middle of the architecture to fuse the multiple receptive fields generated by several max-pooling operators. Lastly, the outputs, generated by three different C3 blocks, are three feature matrixes with different sizes. Traditionally, a non-max suppression algorithm is utilized to analyze the three outputs and calculate the detection boxes according to the preset confidence. In this work, we utilize the output of ResNet50 to guide the calculation of bounding boxes, improving the prediction accuracy of YOLOv5 to the detection boxes.

On the contrary, we add two full connection (i.e., FC) layers at the end of YOLOv5 to calculate the acne severity and number. The architecture is similar to the Linear 1 and 2 blocks in the classification module. Kullback–Leibler divergence loss is adopted to optimize the two blocks. Here, there are two different strategies to train the YOLOv5 and the subsequent classification blocks: (1) Train the YOLOv5 first, and then train the two classification blocks. The output of the trained YOLOv5 is utilized as the input of the two classification blocks. (2) Train the YOLOv5 and two classification blocks simultaneously. The loss of the two classification blocks is added to the loss of YOLOv5, and the total loss function can be written as:

(6) $$\begin{array}{c} loss_{total}=\alpha loss_{YOLOv5}+\beta loss_{classification}, \end{array}$$

where the characters α and β denote the manual coefficients of the losses of the two parts. Specifically, the total loss is composed of two parts, including the loss of the YOLOv5 backbone and classification block, respectively. By adding the two classification blocks into the YOLOv5 backbones, we aim to enable the module to do the acne classification as well as the acne localization simultaneously. Different from method 1, where the two blocks are trained sequentially, a multi-task learning strategy should be adopted to train the modules in method 2. As we all know, different losses guide the model to focus on a different context in the images during training. The localization loss $loss_{YOLOv5}$ here would explore the local geometrical information of each acne, while the classification loss $loss_{classification}$ would force the model to focus more on the global distribution of the acne. Typically, we set the parameters α and β as 0.5 because we want the module could do both equally well in classifying the severity and detecting the acne positions.

## 4. Results

In this section, we first introduce the training parameters and evaluation metrics of the two modules, then detail the inference performance of the classification and localization module discussed in Section 3. Finally, we demonstrate the good performance of the ensemble model in predicting the acne severity, number and position simultaneously.

### 4.1. Training and Evaluation

We train the two neural networks on a single NVIDIA Tesla P100 based on the PyTorch framework. For the classification module, we choose Stochastic Gradient Descent (SGD) with the mini-batch of 32 as the model optimizer. The initial learning rate is set to 0.001 and reduced to half every 30 epochs until it reaches 120 epochs. The momentum and weight decay are 0.9 and $5\times 10^{-4}$, respectively. The input images are resized to 224×224 and normalized by the pre-computed mean and standard deviations. For the localization module, by utilizing the pre-trained YOLOv5 on the COCO datatset, we fine-tune the model with the Adam optimizer and the mini-batch of 32. We set the initial learning rate to 0.0032 and apply a linear attenuation scaling factor from 1 to 0.12 as the epoch increases from 1 to 120. The momentum and weight decay are 0.843 and $3.6\times 10^{-4}$, respectively. Here, the dataset ACNE04 with 1457 images is split into two parts for training (i.e., 80%) and testing (i.e., 20%). Considering that the main purpose here is to predict the acne severity, acne number and acne locations, we select the prediction accuracy and root mean squared error (i.e., RMSE) as the evaluation metrics.

### 4.2. Analyses of Classification and Localization Modules

As discussed in Section 3.2, three different loss functions are adopted to optimize the classification module. Table 1 shows the prediction accuracy of the module trained with cross-entropy loss. Specifically, accuracy_severity and accuracy_number denote the prediction accuracy of the module on the acne severity and number, respectively, and RMSE_count denotes the root mean squared error of the predicted and true acne number. In case 1, we set the rescaling weight $w_{j}$ as the constant (i.e., 1). The module shows high accuracy in predicting the acne severity, while the prediction accuracy of the acne number is very low (<10%), leading to a high RMSE. The main reason is that the distribution of training samples at various acne severity is more uniform than that at acne numbers. In case 2, we consider the imbalance of data distribution and set the weight $w_{j}$ as $n_{j}$, the image numbers in category *j*. The precisions on predicting acne severity and number are increased by about 5% and 250%, respectively. However, the module performance is still far from meeting the medical requirement. Table 2 shows the inference result of the classification module trained with focal loss. In all six cases, the focusing parameter α is 2, inspired by Lin et al. Similar to the two cases in Table 1, we adopt 1 and $n_{j}$ as the weight $w_{j}$ in cases 1 and 2, respectively. In cases 3 and 4, we apply normalization and standardization operation on the manual rescaling weight to reduce the influence of the absolute value of the coefficient on the training. Specifically, normalization is to constrain all values in the sequence to be between 0 and 1 by using the following equation:

(7) $$\begin{array}{c} w_{j}^{{}^{\prime }}=\frac{w_{j}-w_{min}}{w_{max}-w_{min}} \end{array}$$

where $w_{min}$ and $w_{max}$ denote the minimum and maximum value in the coefficient $w_{j}$, respectively. For standardization, the mean and standard deviation of the sequence are used to rescale the sequence:

(8) $$\begin{array}{c} w_{j}^{{}^{\prime }}=\frac{w_{j}-w_{mean}}{w_{std}} \end{array}$$

where $w_{mean}$ and $w_{std}$ denote the mean and standard deviation of the coefficient $w_{j}$. Based on case 3, we additionally require that the sum of all coefficients $w_{j}$ should be 1 to prevent the occurrence of large errors. The inference accuracy of all six cases is given in Table 2. We conclude that using focal loss as the loss function cannot effectively improve the prediction accuracy of the module on acne numbers. Table 3 shows the inference result of the classification module trained with Kullback–Leibler divergence loss. Case 1 is similar to the model provided by Wu et al. [47], while in case 2, we introduce data augmentation discussed in Section 3.1 during training. We find that the RMSE of case 2 is much lower than that of case 1, though case 1 and case 2 show similar prediction accuracy on acne severity and number.

**Figure 3:**
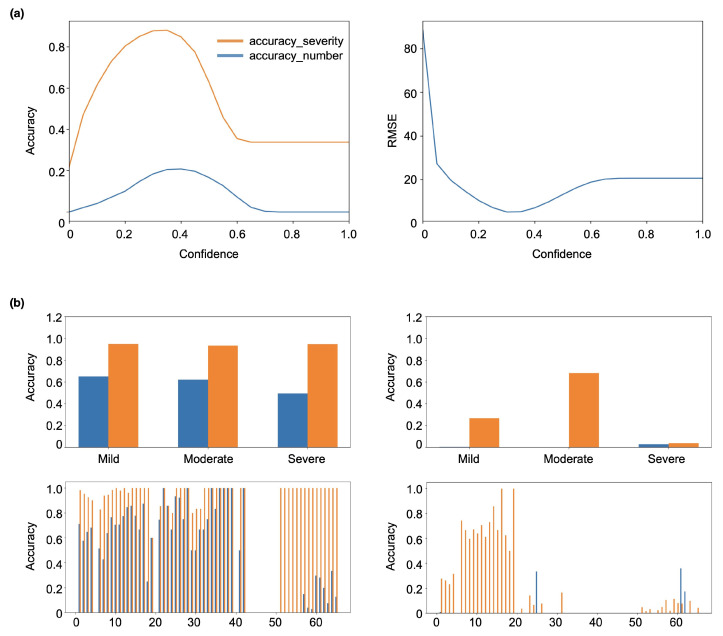
Parametric analyses of localization module. (**a**) The prediction accuracy of YOLOv5 under different confidence values. (**b**) The prediction accuracy of the localization module by adding classification blocks at the end of YOLOv5. In the left panel, the classification block is trained after training the YOLOv5, while in the right panel, the block and YOLOv5 are trained simultaneously.

Figure 3 demonstrates the performance of localization modules with YOLOv5 as the backbone. Since the preset confidence in YOLOv5 is essential to the module output, we have conducted a detailed analysis of the prediction accuracy of the module under different confidences. As shown in Figure 3, the accuracy_severity and accuracy_number first increase and then decrease with the increase of confidences, while the RMSE decreases first and then increases with the increase of confidences. Specifically, when the confidence is around 0.4, the module shows the best performance, where the accuracy_severity, accuracy_number and RMSE are about 0.85, 0.2 and 7, respectively. We find that the YOLOv5 is not suitable for predicting the acne number. Since the output of YOLOv5 usually includes the coordinates of the objects and the corresponding probability of confidence, it is hard to use a unified confidence probability to accurately assess all categories of acne number and severity in the application of acne detection. The main reason is that facial acne shows a similar background to other facial features, such as the nose and mouth contour, and can be easily affected by the ambient light and photograph angle. Then, we add classification blocks composed of fully connected layers to resolve this problem. As shown in Figure 3b, we adopt two different training strategies discussed in Section 3.3. The left and right panels are the inference result of training methods 1 and 2, respectively. We find that training method 1 is much better than training method 2. The main reason is that the simultaneous training of the two blocks will cause the noise between different blocks to interfere with each other, while sequential training would limit the noise in each block. However, when the acne number is large, the module performance becomes poor, which is still far from meeting the medical requirement.

We also compare our methods with previously reported work (as shown in Table 3). Specifically, two classical detection models, i.e., the Faster RCNN and YOLOv3, are adopted to count the acne number in the facial image [47]. The best prediction accuracy of the two models is 73.97% and 64.70%, respectively, which is much lower than our methods here. The main reason for the high accuracy in severity classification is that we smooth the data and reclassify the labels of the dataset into three acne grades. In the classification of four acne grades, the AcneGrader [52] proposed by Liu et al. shows a higher prediction accuracy (>85%) on the ACNE04 dataset and outperforms state-of-the-art methods. Besides, the minimum RMSE between the true and predicted numbers here is only 2.17, which is about 35.61% lower than the error in F-RCNN and YOLOv3 model. Note that our methods here show a comparable accuracy to that of Wu et al. However, the previous models cannot calculate the acne positions in the facial image. In this paper, we can not only accurately predict the acne number and severity, but can also predict the location of facial acne, assisting doctors in acne diagnosis. In conclusion, the main advantages of the proposed model here lie in two aspects: (1) We smooth the dataset by reclassifying the datasets into three categories and utilize random sampling methods to preprocess the input images, improving the prediction accuracy of acne number and severity; (2) We introduce modified YOLOv5, which is controlled by the classification features to calculate the acne position in the facial images.

## 5. Discussion

**Figure 4:**
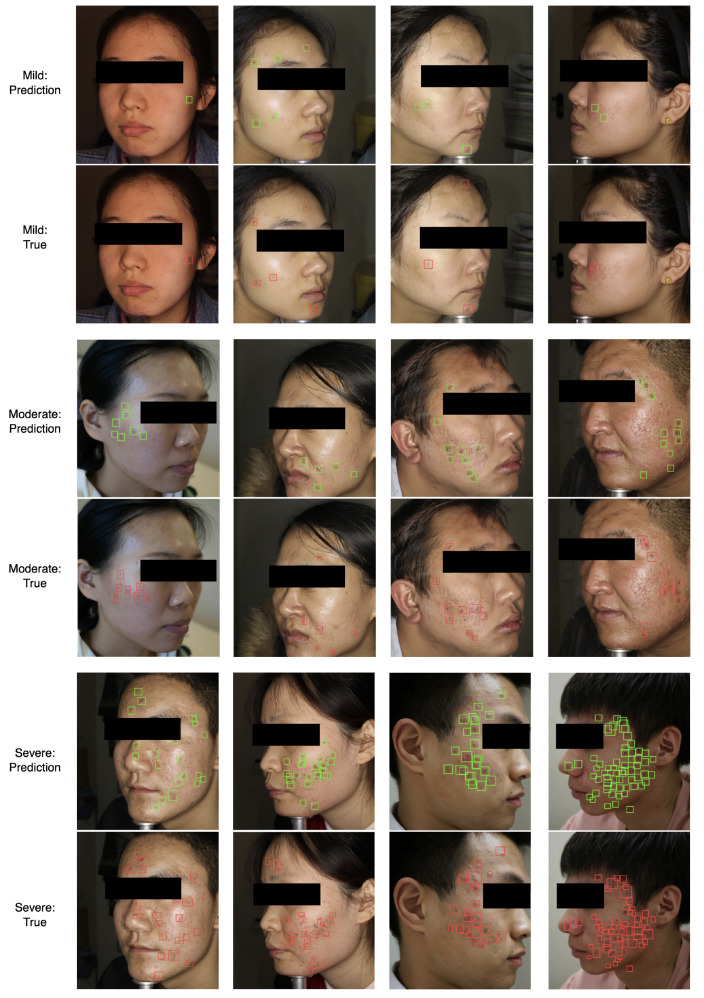
Representative examples of the true results and prediction images generated by the ensemble model.

**Figure 5:**
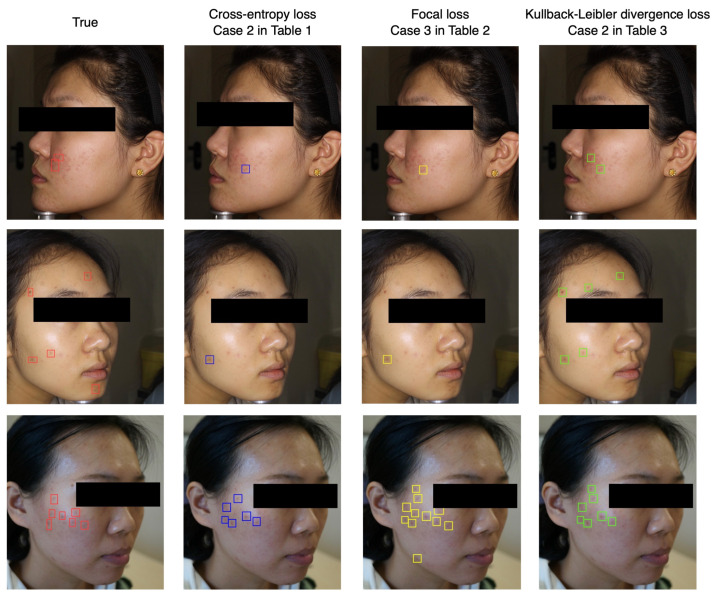
The effect of different loss functions (such as the cross-entropy loss, focal loss and Kullback–Leibler divergence loss) on acne detection.

From the previous parameters studies, we find that the localization module shows relatively poor performance on the prediction of acne number, although the module could detect the acne in the facial image. Considering that the classification module based on ResNet50 could calculate the acne severity and number precisely after training with Kullback–Leibler divergence loss, we combine the classification and localization modules into the ensemble model to enhance the precision of detecting acne. As shown in Figure 1, the output of the Linear 2 block in the classification module is connected to the input of the detect block in the localization module. Instead of the preset confidence, we utilize acne numbers to control the output of detection boxes. Figure 4 shows several examples of the ensemble model. The top, middle and bottom panels correspond to the mild, moderate and severe classes, respectively. In each panel, the upper and lower rows represent the predicted and true results of each class. We find that the predicted results agree well with the real results and the acne severity, number and position can be achieved simultaneously. We also compare the effect of different loss functions on acne detection. As shown in Figure 5, the right three columns are the prediction result of the models trained by cross-entropy loss, focal loss and Kullback–Leibler divergence loss, corresponding to the cases (i.e., case 2, case 3 and case 2) with the lowest RMSE in Table 1, Table 2 and Table 3, respectively. We find that the model trained by cross-entropy loss and focal loss show a large error in predicting the distribution of acne during inference. The main reason is that the Kullback–Leibler divergence loss could force the model to learn the probability distribution of the acne numbers instead of the category index, greatly improving the model’s capability of detecting facial acne. It is worth mentioning that when the distribution density (e.g., >50 per image) of acne in the face is very high, the model here would show a poor performance in assessing the acne number and locations.

## 6. Conclusions

This paper proposes a novel ensemble model to detect facial images, including calculating the acne severity, number and position. The model consists of two submodules: (1) the classification module used to calculate the acne severity and number and provide guidance for the inference of the localization module; (2) the localization module used to calculate the detection boxes under the assistance of the classification module. This is the first time that the acne severity, number and position are simultaneously predicted through deep learning, and the prediction results show good agreement with the true results. Furthermore, considering that the acne in the different body parts (such as the face and back) usually shows similar geometrical configurations (i.e., a cone with the apex approximately in the center), the proposed model can be further applied in detecting back acne, chest acne, etc. This method could assist patients with self-testing by taking a selfie according to the Hayashi grading criterion, and help doctors diagnose acne problems. This ensemble model will show significant applications in medical engineering.

## Figures and Tables

**Table 1:** The prediction error of the classification module, which is trained by using cross-entropy loss of different rescaling weights to each acne severity value and acne number.

	Accuracy_Severity	Accuracy_Number	RMSE_Count
Case 1	90.67%	6.18%	10.54
Case 2	95.06%	21.48%	9.07

**Table 2:** The prediction error of the classification module, which is trained by focal loss with different parameters.

	Accuracy_Severity	Accuracy_Number	RMSE_Count
Case 1	99.45%	25.88%	11.70
Case 2	43.44%	11.81%	19.91
Case 3	99.45%	16.00%	7.93
Case 4	21.35%	0%	34.90
Case 5	99.45%	14.76%	8.42

**Table 3:** The prediction error of the classification module, which is trained by Kullback–Leibler divergence loss with/without data augmentation.

	Accuracy_Severity	Accuracy_Number	RMSE_Count
Case 1	99.31%	84.60%	2.74
Case 2	99.17%	84.17%	2.17
F-RCNN		73.97%	3.39
YOLOv3		63.70%	3.37
Wu et al.		84.11%	2.33

## Data Availability

Data underlying the results presented in this paper are available by contacting the first author or the corresponding author.
